# Editorial: Advances in orally inhaled and nasal drug products (OINDPs)

**DOI:** 10.3389/fphar.2023.1185609

**Published:** 2023-03-31

**Authors:** Piyush Pradeep Mehta, Eleonore Fröhlich, Raju Khan, Arpana Parihar, C. M. Santosh Kumar

**Affiliations:** ^1^ Cipla R&D Center (Vikhroli), Mumbai, India; ^2^ Center for Medical Research, Medical University of Graz, Graz, Austria; ^3^ CSIR - Advanced Materials and Processes Research Institute (AMPRI), Bhopal, India; ^4^ Institute of Microbiology and Infection, School of Biosciences, University of Birmingham, Birmingham, United Kingdom

**Keywords:** dry powder inhaler, pulmonary delivery, nasal formulations, nanocrystal, electro-spraying, amiodarone

## Advances in orally inhaled and nasal drug products (OINDPs)

Orally Inhaled and Nasal Drug Products (OINDPs) are of growing importance in the pharmaceutical domain, owing to their intrinsic benefits over the traditional dosage forms, such as low first pass effect by hepatic metabolization, fast onset action, and high drug levels in brain tissue (nasal route) and lung (oral inhalation). Moreover, OINDPs can treat both local (e.g., asthma and COPD) and systemic (e.g., Parkinson’s disease and diabetes) diseases. One of the efficient delivery mechanisms for the substances, such as synthetic drugs, phytoconstituents, genes, proteins, and peptides is by inhalation through the pulmonary airways. Therefore, OINDPs present potential approaches to expand pharmaceutical commercialization opportunities. In this thematic issue, we have judiciously selected original research and review articles that build a strong foundation for understanding the myriad of applications of the OINDPs. The thematic issue contains one review article and three research articles on OINDPs. The thematic issue begins with a comprehensive review of the recent advancements in nasal formulations. The review article by Dhapte-Pawar and co-workers summarizes the key developments in nano-carrier-based nasal drug delivery systems for effective neurotherapeutic molecule delivery (Rajput et al., 2022). The article discussed various nano-carriers, such as solid lipid nanoparticles, nanostructured lipid carriers, lipid drug conjugates, micro/nanoemulsions, lipid vesicles, carbon nanotubes, dendrimers, and micelles, with a special importance on preclinical and clinical (phase 1 and 2) outcomes. Further, the current challenges and opportunities for future development of novel nasal nano-carriers are also presented to accelerate their early clinical uses.

Punna Rao Ravi and team present the physicochemical characterization and animal testing data of an *in situ* thermal responsive nasal gel, containing rufinamide (an anticonvulsant medication) in epilepsy treatment (Dalvi et al., 2022). The nanocrystal-based formulation markedly increased rufinamide levels in the rat plasma and brain, than when administered intranasally. The results obtained from the physicochemical and pre-clinical investigation suggest that nasal delivery of nanocrystals is beneficial in enhancing the therapeutic outcome.

David and co-workers proposed a local treatment regimen for bronchial carcinoma by cisplatin electro-spraying (Ruzgys et al., 2021). The authors studied the efficacy of electrosprayed cisplatin *in-vitro* by administering it to Lewis lung carcinoma cells and *in vivo* by using this technique on subcutaneously implanted Lewis lung carcinoma cells in C57BL/6J mice. They observed decreased viability and apoptosis induction in the cellular studies and decreased tumor size and an increased number of apoptotic cells *in vivo*. Briefly, this proof-of-concept study showed that targeted drug delivery by electrospray is efficient in lung cancer management and presents a potential clinical application.

Kaneko and team highlight the negative impact of administering higher doses of a pulmonary drug, by focusing on drug-induced interstitial lung disease (Siswanto et al., 2021). This pathology is characterized by inflammation, fibrosis, and respiratory insufficiency. One of the most frequent inducers of this pathology is the anti-arrhythmic drug amiodarone. The authors reviewed the FDA Adverse Event Reporting System and JMDC Inc. insurance claims to identify a coexisting drug that reduced the incidence of ILD associated with the application of amiodarone. Based on these data, the authors identified a thrombin inhibitor dabigatran as a potential protectant against ILD. The authors validated the hypothesis in amiodarone treated C57BL/6J mice. Inhibition of matrix metalloproteinases and transformation of fibroblasts to myofibroblasts by dabigatran appears to be more important for protection than anticoagulant action.

The manuscripts in this research topic describe some of the possibilities for pulmonary and intranasal administration. Other promising applications for pulmonary and intranasal delivery include pain treatment and mucosal vaccination. The ease-of-production of the nasal formulations and their unique access to the brain make intranasal administration more attractive than oral inhalation in systemic delivery. The major limitations of the nasal route are the smaller absorption area, the minimal volume of the administered formulation, the dilution of the drug by mucosal secretions, the potential mucosal irritation upon chronic use, and the shorter retention. Methods to increase drug concentrations of intranasal formulations are likely to help in overcoming some of these limitations (Pires et al., 2022). This thematic issue focused on the OINDP’s formulations. However, there is an appealing story to be told regarding particle engineering, automated particle imaging, realistic mouth-throat modeling, computational fluid dynamics, inhaler designing, and patient training.

We would like to thank all the contributors to this thematic issue for their valuable time and contributions. We hope that these review and research articles offer a valuable reference for the researchers to probe the role of OINDPs to persist in the important battle against life-threatening pulmonary diseases.
